# Psychological and Social Factors Associated with Coexisting Frailty and Cognitive Impairment: A Systematic Review

**DOI:** 10.1177/01640275211045603

**Published:** 2021-10-02

**Authors:** Alison Ellwood, Catherine Quinn, Gail Mountain

**Affiliations:** 1 Centre for Applied Dementia Studies, 1905 University of Bradford, Bradford, UK; 2 Wolfson Centre of Applied Health Research, Bradford, UK

**Keywords:** dementia, older people, depression, anxiety, education

## Abstract

Those living with coexistent frailty and cognitive impairment are at risk of poorer health outcomes. Research often focuses on identifying biological factors. This review sought to identify the association psychological and social factors have with coexisting physical and cognitive decline. Six databases were systematically searched in July 2020. Studies included individuals aged 60 years or older identified as being both frail and cognitively impaired. A narrative synthesis examined patterns within the data. Nine studies were included, most employed a cross-sectional design. Depression was investigated by all nine studies, those with coexistent frailty and cognitive impairment had higher levels of depressive symptoms than peers. Findings were mixed on social factors, although broadly indicate lower education, living alone and lower material wealth were more frequent in those living with coexistent decline. Further research is needed to explore potentially modifiable psychological and social factors which could lead to the development of supportive interventions.

## Introduction

Increases to life expectancy have led to elevated risk of age-related illness and associated disability (Lunenfeld & Stratton, 2013). Therefore, ensuring healthy ageing is a priority for both healthcare providers and policy makers worldwide (WHO, 2015). This includes improved understanding of factors linked to age-related illness and improving healthcare provision for older people (WHO, 2015). Linked to this there is increasing interest in the concept of biological ageing as there is evidence that some individuals appear to develop age-related ill health earlier than others (Vernon, 2020). Differences in the occurrence of age-related ill health has been linked to the accumulation of deficits, such as symptoms, disease states and functional impairments, in later life a concept strongly linked with frailty (Mitnitski et al., 2013). Social inequalities and psychosocial factors may play a role in this chronological discrepancy given there is an established association with poor health outcomes across the life course and into old age (Braveman & Gottlieb, 2014). To address these inequalities in age-related health, the focus has been on identifying factors linked to poorer outcomes that might be amendable by intervention to promote healthy ageing. Consequently, one area of interest is the identification and support of those living with frailty (Vernon, 2020).

Frailty syndrome has been conceptualized as a decline over multiple biological systems, resulting in diminished resistance and capacity to return to former functioning following stressor events (Clegg et al., 2013). Frailty is associated with, yet distinct from, ageing, comorbidity and disability (Fried et al., 2001). Two major approaches to measuring frailty persist. The phenotype of frailty includes physiologic components only. These are unintended weight-loss, poor endurance, reduced physical activity, slow gait and weak grip (Fried et al., 2001). Alternatively, the accumulated deficits model implicates the build-up of symptoms, disease states, abnormal test results and disabilities in an index (Mitnitski et al., 2001). This model is more likely to include cognitive, psychological and social aspects. There is no consensus over the best approach to identifying those with frailty; however, both the phenotype criteria and frailty indices are broadly accepted by clinicians (de Vries et al., 2011). Frailty has been linked to adverse outcomes such as increased disability, hospitalization, admission to long-term care and death (Clegg et al., 2016; Fried et al., 2001; Theou et al., 2013). While frailty is long-term and progressive, it has been proposed that the state is modifiable with appropriate support and timely intervention (Travers et al., 2019).

Increasing age is linked to elevated risk of frailty; similarly, changes to cognition are also seen to increase with ageing. Although some cognitive change is anticipated with normal ageing, pathological decline can lead to impaired cognitive function and dementia (Jessen et al., 2014). There appears to be an association between frailty, as captured by the phenotype criteria, and cognitive impairment (Fried et al., 2001). Exploration of the relationship between frailty and impaired cognition indicates there may be shared biological mechanisms between the two (Panza et al., 2006), although as yet this is poorly understood (Kant et al., 2018). There have been attempts to describe this link further, including the conceptualization of cognitive frailty (Kelaiditi et al., 2013). Cognitive frailty is defined as the presence of both frailty and cognitive impairment, rated as questionable dementia (0.5) on the Clinical Dementia Rating (CDR) scale (Hughes et al., 1982). This definition excludes those living with dementia. One of the key aims, in associating cognition with frailty, was to identify reversible cognitive decline in old age, given the proposal that frailty is treatable and modifiable. Studies have established an association between frailty and specific cognitive domains such as executive function (Bunce et al., 2019) and motoric cognitive risk, which associates cognitive decline with slow gait, a measure of the frailty phenotype (Montero-Odasso et al., 2016). These findings indicate a relationship between cognition and physical function, although the exact causal route is uncertain.

Both frailty and impaired cognition are individually associated with adverse health outcomes and poor quality of life (Lim et al., 2018; Lucke et al., 2018; Malmstrom et al., 2014). Furthermore, an accumulative effect of frailty and cognitive impairment is apparent. Those with coexistent frailty and cognitive impairment experience more hospitalizations, more frequent admission to long-term care and a more rapid decline to dependency and death, than healthy peers or those living with frailty or cognitive impairments alone (Avila-Funes et al., 2009; Feng et al., 2016; Panza et al., 2018). Therefore, given the negative outcomes associated with having both frailty and cognitive impairment, it is important to understand the factors that increase an individual’s risk of having coexistent frailty and cognitive impairment in later life. Moreover, it is particularly important to identify modifiable factors which have been linked to both frailty and cognitive impairment, to aid in the development of targeted therapy and support.

The association frailty coexistent with cognitive impairment has with biological factors is well investigated. Clinical markers such as cardiovascular disease and elevated blood pressure or evidence of physiologic change such as chronic inflammation or hormonal changes have been examined (Sargent et al., 2018). However, there may be risk factors that occur earlier in the lifespan. Longitudinal evidence indicates that multiple factors, including those of a social or psychological nature, which have been given far less consideration in research thus far, may present as risks, or provide protection, against frailty (Feng et al., 2017). The need to look at other health determinants, beyond physiology, is implicated in the search for modifiable factors and therapeutic measures. Psychological factors such as depression (Soysal et al., 2017), and social factors such as level of education (Ng et al., 2014), income (Marshall et al., 2015), living arrangement (Makizako et al., 2018) and marital status (Peek et al., 2012) have all been identified as being associated with frailty. Similarly, there is also evidence that psychological and social factors influence the onset and/or trajectory of cognitive decline in older people (da Silva et al., 2013; Kuiper et al., 2015). This suggests that psychological and social factors potentially contribute to vulnerability associated with coexistent frailty and cognitive impairment, warranting further exploration, particularly as these are potentially modifiable factors.

While under-researched in comparison with biological factors, psychological and social factors are being increasingly considered in relation to coexistent frailty and cognitive impairment (Kwan et al., 2019; Li et al., 2020; Rivan et al., 2020). Yet, no previous review has explored the influence of psychological and social factors on this coexistent decline. Previous reviews have focussed on biological risk factors or health outcomes (Facal et al., 2019; Sugimoto et al., 2018). Identification of factors which pose risks for developing coexistent frailty and cognitive impairment, and those which might offer protection, is vital in developing appropriate therapies and supportive interventions. Increased awareness of factors which contribute to coexistent decline may improve treatment suitability for older people living with coexistent frailty and cognitive impairment.

Aim: The aim of this review is to identify, and evaluate the quality of, existing evidence regarding the nature and impacts of psychological and social factors in people living with coexistent frailty and cognitive impairment.

## Methods

### Search Strategy and Registration

The review was carried out in accordance with the Preferred Reporting Items for Systematic Reviews and Meta-Analyses (PRISMA) criteria (Page et al., 2021). The search strategy included a systematic search of Medline, CINAHL, Embase, AMED, PsychINFO and Cochrane databases (from January 2001 to July 2020 inclusive). Three key search concepts included ‘frailty’, ‘cognitive impairment’ and ‘psychosocial factors’ with a wide range of associated keywords. These were combined in a full text search to enable a comprehensive and inclusive examination of the existing literature. Table 1 gives an example of the search strategy; modifications were made to implement a comparable search in all databases. Additional searches were conducted in EThoS, NTLTD and Proquest Express to identify theses. Forward and backward citation searching was used to identify additional studies from relevant retrieved papers. The review protocol was registered with PROSPERO: CRD42020196086.

**Table 1.** Search Term Example: Medline.

Concept	Theme	Terms
1	Frailty	frai*; “gait speed”; “sarcopenia”; “frailty index”; “clinical frailty scale”; “motoric cognitive risk” π
2	Cognitive impairment	cogniti*; mci; “mild cognitive impairment”; “memory impairment”; “memory decline”; cind; “cognitive impairment no dementia”; dementia; Alzheimer*; “motoric cognitive risk” π
3	Psychological and social factors	“social frailty”; Socioeconomic*; “social network”; “social support”; “social environment”; “social environment”; “social vulnerabilit*”; “social contact”; “marital status”; “living alone”; income; educat*; loneliness; isolat*; neighb#rhood; psychology*; depressi*; wellbeing; well-being; “quality of life”; “low mood”; anxiety; “mental health”; “negative affect”; “positive affect”; “self-rated health”; psycho-social; psychosocial

π Motoric cognitive risk combines both concept 1 and concept 2.

Search combines concepts 1 AND 2 AND 3.

Limited to publications in English from January 2001 to July 2020 inclusive.

### Inclusion and Exclusion Criteria

The search was limited to articles written in English published after 2001, when the landmark Cardiovascular Health Study paper (Fried et al., 2001) was published which defined and identified criteria for the measurement of frailty in older people. Although age associated decline cannot be determined by chronological age, and there is global variation, the World Health Organisation considers 60 as a global threshold for old age (WHO, 2018). As such, this age limit was used as an inclusion criterion within this review. We included only studies which used primary data with the additional criteria to identify comparable studies:

### Inclusion Criteria

- Studies involving individuals aged 60 years or older identified as being both frail and cognitively impaired.
- Where frailty is measured using a standardized measure of either the phenotype approach (Fried et al., 2001) or alternatively using a frailty index which has no cognitive, psychological or social components and developed using pre-defined set criteria (Searle et al., 2008).
- Where cognitive impairment is defined as an impairment which is more severe than that which is associated with normal ageing, assessed using self-report or validated measures or clinician diagnosis.
- Studies exploring factors that were either psychological (e.g. depression, self-esteem or optimism) or social (e.g. income, education, living arrangement, marital status or social support) in nature.
- Studies using randomized, quasi-randomized, cohort and case-controlled designs.

### Exclusion Criteria

• Studies where data on those aged ≥60 years cannot be extracted.
• Studies that did not include people with both frailty and cognitive impairment, or this was ambiguously defined or measured.

### Review Process

**Figure 1:**
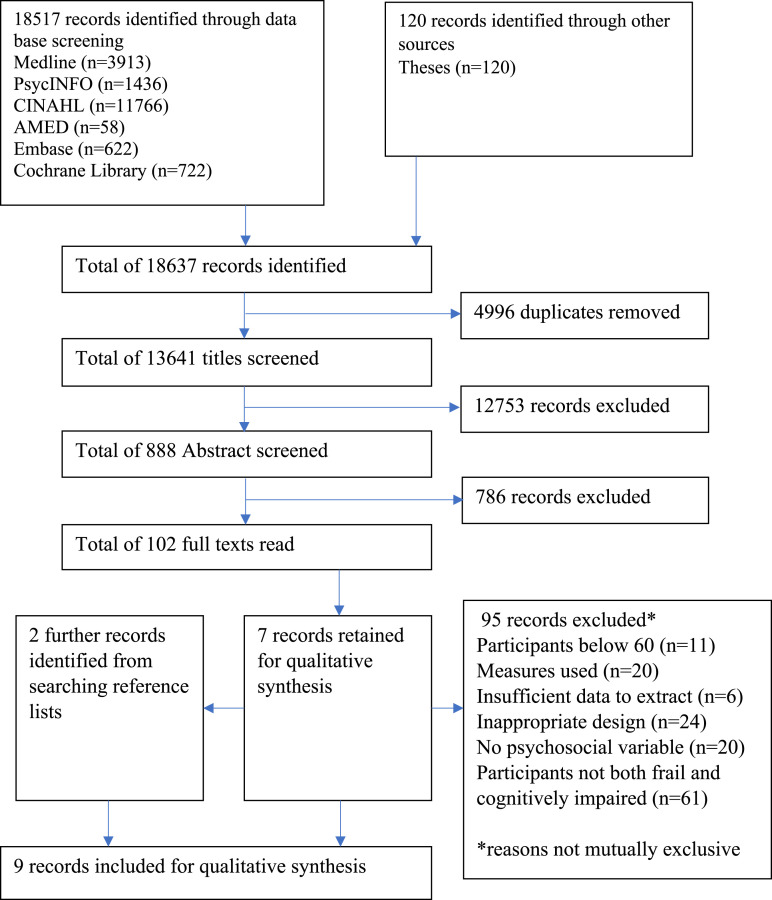
PRISMA flow chart of records through the screening process.

Figure 1 illustrates the literature search process. EndNote Software version X9 was used to facilitate screening. Title, abstract and full text screening was conducted by two reviewers. There was 94.0% agreement on title screening, 81.3% agreement on abstract screening and 93.7% agreement on full text screening. Any disagreements were resolved through discussion until consensus was achieved.

### Data Extraction

For each paper included in the review, information was collected on study design, county, year of data collection, year of publication, the setting, characteristics of the sample (including sample size, age, gender and ethnicity), the instrument to measure frailty, the measure of cognitive impairment and the measure(s) of psychological and social factors. In addition, the main significant associations were extracted. It was noted during extraction that some studies used different thresholds for significance; in this review, a *p* value of 0.05 was used for the purposes of comparability. Data were extracted by one reviewer and a proportion of papers reviewed by a second.

### Study Quality Assessment

Given the heterogeneity of the study designs, quality was assessed using a tool devised to reflect the diversity of analytical approaches included in the review, the 16-item quality assessment tool (QATSDD) (Sirriyeh et al., 2012). Quality was assessed using the following criteria: clear aims, objectives and theoretical basis for the study, sample size rationalized and methods for recruitment and data collection stated and rationalized, analytical methods befitting the research question and evidence of reflection on the study limitations. Each criterion was rated on a four-point scale, with higher scores indicating higher quality, up to a total of 42. Quality assessment was undertaken by two reviewers, and disagreements were resolved through discussion. Studies which were assessed as lower in quality were still included in the review due to the emergent nature of the field, the low number of studies and overall quality.

## Results

### Study Selection

Figure 1 summarizes study selection. A total of 18,637 records were identified, and 102 were retrieved for full text reading and nine were included for data extraction. The main reasons for excluding studies upon reading the full text were that participants were not identified as both frail and cognitively impaired (*n* = 61) and the design or analysis was inappropriate for inclusion (*n* = 24) (see Figure 1).

### Study Characteristics

Characteristics of the nine included studies are shown in Table 2. All studies were published between 2016 and 2020, with the majority published since 2019. A total of 24,617 older adults were included. The majority of studies were conducted in Asia, in China (Ge et al., 2020; Kwan et al., 2019), Taiwan (Li et al., 2020; Wu et al., 2020), Malaysia (Rivan et al., 2020; Rivan et al., 2019) and Japan (Shimada et al., 2016). One was conducted in the United States (Aliberti et al., 2019) and one in Mexico (Aguilar-Navarro et al., 2019). Studies were undertaken with those living in the community, although recruitment was through hospital clinics in two studies (Aguilar-Navarro et al., 2019; Wu et al., 2020). There was considerable heterogeneity in study designs (see Table 3). Three studies involved cross-sectional data analysis of cohorts (Aliberti et al., 2019; Rivan et al., 2019; Shimada et al., 2016). One employed a longitudinal cohort design (Rivan et al., 2020). All studies included a population with both physical and cognitive decline with at least one comparison group, and from this they produced prevalence estimates. The reported prevalence of frailty coexistent with cognitive impairment in the study populations was highly variable ranging from 1.2% to 39.6% (Table 3). The majority of studies included three comparison groups, either healthy peers, or those with frailty or cognitive impairment alone (Aguilar-Navarro et al., 2019; Aliberti et al., 2019; Ge et al., 2020; Kwan et al., 2019; Li et al., 2020; Shimada et al., 2016; Wu et al., 2020). Although Aguilar-Navvarro et al. (2019) had three comparison groups, these were used inconsistently in analyses. Rivan et al. (2019) compared those with coexistent frailty and cognitive impairment with only healthy individuals. While in the study by Rivan et al. (2020), the comparison group was less clearly defined and simply described as those who had not developed both frailty and cognitive impairment. Therefore, was likely comprised of a mixed group of healthy individuals and those with frailty or cognitive impairment alone (see Table 3).

**Table 2.** Characteristics of Included Studies (n = 9).

First Author	Publication year	Data collection year(s)	Country	Study setting	Total sample size all participants baseline (and follow up)	Mean age of total sample all participant (±SD)	Gender % female	Quality assessment
Aguilar-Navarro	2019	2017-2018	Mexico	Memory clinic	180	72.99 ±6.6	75% female	24
Aliberti	2019	2006-2008	USA	Community	7338	74.4 ±7.0	54.9% female	29
Ge	2020	2018	China	Community	4103	67.8 ±5.9	58.3% female	27
Kwan	2019	2018-2018	China	Community	185	86.2±4.5	71.4% female	24
Li	2020	2013	Taiwan	Community	2693	Not stated	Not stated	23
Rivan	2019	2012	Malaysia	Community	815	68.86 ±6.12	54.4% female	29
Rivan	2020	2017	Malaysia	Community	490 baseline, 282 Follow-up	Not stated	Not stated	29
Shimada	2016	2011-2013	Japan	Community	8864	73.4±5.4	52.0% female	27
Wu	2020	2018	Taiwan	Geriatric clinic	157	79.4±7.9	66.9% female	20

**Table 3:** Measures of Included Studies (n = 9).

First Author, date	Study design	Frailty assessment	Cognitive assessment(s)	Defining mixed group	Comparison groups	Prevalence comorbid group in study population	Psychological factors and assessment methods	Social factors and assessment methods
Aguilar-Navarro, 2019^e^	Cross-sectional	Fried Phenotype^a^	MCI^c^ MRI MMSE NEUROPSI	Fried 3 or more criteria^a^, MCIv^c^	n = 1 healthy	17%	Depressive symptoms (GDS-15)	None
Aliberti, 2019	Cohort	Fried Phenotype^a^	HRS validated approach 27-point scale: normal = 12-27; CIND = 7-11; dementia ≤6	Fried 3 or more criteria^a^, CIND	n = 3 healthy, frail only CIND only	5%	Depression (CES-D ≥3 symptoms classed as depression)	Married (yes or no Education level) (completed high school or not) Net worth (total household assets minus current debt)
Ge, 2020	Prospective observational study	Fried Phenotype^a^	SPMSQ adjusted for education level (+2 for those with high school education or +4 for those with primary education only or lower)	Fried 3 or more criteria, cognitive impairment on SPMSQ^a^	n = 3 healthy, frail only, cognitive impairment only	2.9%	Depression (GDS-15 ≥8 symptoms classed as depression)	None
Kwan, 2019	Cross-sectional	FRAIL Scale^b^	MCI^c^ MMSE	FRAIL 1 or more criteria^b^, MCI^c^	n = 3 healthy, frail only, MCI only^c^	35.7%	Depressive symptoms (GDS-15)	Education level (primary or below, secondary, tertiary or above)
Li, 2020	Cross-sectional	FRAIL Scale^b^	MMSE adjusted for education level (24/30 for 7+years education, 20/30 for 1-6 years of education, 17/30 if illiterate)	FRAIL 1 or more criteria^b^, cognitive impairment on MMSE	n = 3 healthy, frail only, cognitive impairment only	11%	The presence of anxiety and depression (EQ-5D answers dichotomised yes/no)	Education level (illiterate, 1-6 years, ≥7 years) Marital status (married/living with partner or not)
Rivan, 2019^d^	Cohort, cross-sectional	Fried Phenotype^a^	MCI^c^ Digit Span or RAVLT MMSE	Fried 1 or more criteria^a^, MCI^c^	n = 1 healthy	39.6%	Depressive symptoms (GDS-15)	Education level (≤ 6 years or >6 years) Occupation (working or not) Household income (high or low) Marital status (married or not) Social support (MOS-SS)
Rivan, 2020^d^	Cohort, longitudinal	Fried Phenotype^a^	MCI^c^, Digit Span or RAVLT MMSE	Fried 1 or more criteria^a^, MCI^c^	n = 1 healthy	35.5%	Depressive symptoms (GDS-15)	Education level (years) Living alone status (yes/no) Household income
Shimada, 2016	Cohort	Fried Phenotype^a^	NCGG-FAT 4 domains cognitively impaired if 2 or more limitations	Fried 3 or more criteria^a^, 2-4 limitations on NCGG-FAT	n = 3 healthy, frail only, cognitive impairment only	1.2%	Depressive symptoms (GDS-15)	Education Level (years) Living alone status (yes or no)
Wu, 2020	Cross-sectional	FRAIL Scale^b^	CDR = 1-2 mild to moderate dementia MMSE 10-24	FRAIL 3 or more criteria^b^, CDR = 1-2 mild to moderate dementia MMSE 10-24	n = 2 cognitive impairment only, cognitive impairment and pre-frailty	15.9%	Depression (GDS-15 ≥6 points classed as Depression)	Marital status, (Married or not) Living condition (living with family or not) Education Level (years)

^a^Fried (Fried et al. 2001) frailty phenotype 5 criteria for measurement: weight-loss, poor endurance, reduced physical activity, slow gait, and weak grip. 0 criteria classified as healthy, 1 or 2 criteria classified as pre-frail, 3 criteria classified as frail, based on the Cardiovascular Health Study criteria, studies use the criteria and base cut offs around gender and age specific cut offs

^b^FRAIL Scale (Morley et al. 2012): Fatigue, Resistance, Ambulation, Illness, Loss of weight) frailty 3-5, pre-frailty 1-2; healthy 0

^c^MCI: Mild Cognitive Impairment by Petersen Criteria (Petersen 2004) CDR = 0.5 (Berg 1984); intact functional activities; subjective memory complaint

^d^Rivan 2019 and Rivan, 2020 used the same cohort, one as a cross-sectional study the other as longitudinal.

^e^Aguilar-Navarro 2019 had 3 control groups, however when comparing groups on depressive symptoms they compared the mixed group and healthy group.

MRI: Magnetic Resonance Imaging; MMSE: Mini-Mental State Examination; CDR: Clinical Dementia Rating scale; NEUROPSI: neuropsychological evaluation in Spanish; MCIv: Mild Cognitive Impairment with vascular aetiology; GDS-15: Geriatric Depression Scale-15 item; HRS: Health and Retirement Study; CIND: Cognitive Impairment No Dementia; CES-D: Center for Epidemiologic Studies Depression Scale; SPMSQ: Short Portable Mental Status Questionnaire; EQ-5D: EuroQol-5 dimension; RAVLT: Rey Auditory Verbal Learning Test; NCGG-FAT: National Center for Geriatrics and Gerontology Functional Assessment Tool; MOS-SS: Medical Outcomes Study Social Support Survey.

### Methodological Quality of Included Studies

The quality of studies was moderate with scores ranging from 20 to 29 out of a possible 42 (see Table 2). The approach to data collection and analysis was generally of a high standard; however, there were no theoretical considerations described in any included study. In addition, there was no described involvement from those living with frailty and cognitive impairment in the conception or design of the studies.

### Measures of Frailty and Cognitive Impairment

Frailty was evaluated with relative consistency across the studies. The Cardiovascular Health Study criteria (Fried et al., 2001) were used in six studies (Aguilar-Navarro et al., 2019; Aliberti et al., 2019; Ge et al., 2020; Rivan et al., 2020; Rivan et al., 2019; Shimada et al., 2016) (see Table 3). With this definition frailty is measured through weight-loss, poor endurance, reduced physical activity, slow gait and weak grip. Participants were classified as frail if they meet any three, or more, criteria and pre-frail if they meet one or two. However, studies did vary in how they collected this data and applied established population appropriate cut-off points. The remaining three studies used the FRAIL scale (Morley et al., 2012) (Kwan et al., 2019; Li et al., 2020; Wu et al., 2020) collecting data on fatigue, resistance, ambulation, illness and loss of weight. For this scale having three or more criteria is classified as frail and pre-frail if there was evidence of one or two.

There was greater variation in how cognitive impairment was measured by the studies (see Table 3). Five studies used multiple assessments to establish that participants were cognitively impaired (Aguilar-Navarro et al., 2019; Kwan et al., 2019; Li et al., 2020; Rivan et al., 2020; Rivan et al., 2019). Assessments included the Mini-Mental State Examination (MMSE) (Folstein et al., 1975) (Aguilar-Navarro et al., 2019; Kwan et al., 2019; Li et al., 2020; Rivan et al., 2020; Rivan et al., 2019; Wu et al., 2020); Short Portable Mental Status Questionnaire (SPMSQ) (Pfeiffer, 1975) (Ge et al., 2020); National Centre for Geriatrics and Gerontology-Functional Assessment Tool (NCGG-FAT) (Makizako et al., 2013) (Shimada et al., 2016); Rey Auditory Verbal Learning Test (RAVLT) (Schmidt, 1996) (Rivan et al., 2020; Rivan et al., 2019); Digit Span Forward and Backward test (Rivan et al., 2020; Rivan et al., 2019); neuropsychological evaluation in Spanish (NEUROPSI) (Ostrosky-Solís et al., 2007) (Aguilar-Navarro et al., 2019); Health Retirement Study (HRS) (Sonnega et al., 2014) validated assessment (Aliberti et al., 2019), diagnostic criteria such as Petersen criteria for MCI (Petersen, 2004) (Aguilar-Navarro et al., 2019; Rivan et al., 2020; Rivan et al., 2019) and/or the Clinical Dementia Rating scale (Berg, 1984) (Aguilar-Navarro et al., 2019; Kwan et al., 2019; Wu et al., 2020) and neuroimaging techniques Magnetic Resonance Imaging (MRI) (Aguilar-Navarro et al., 2019). Two studies adjusted cut-off points in line with the educational level of participants for the SPMSQ (Ge et al., 2020) and the MMSE (Li et al., 2020). Four studies (Aguilar-Navarro et al., 2019; Kwan et al., 2019; Rivan et al., 2020; Rivan et al., 2019) used subjective memory complaints as part of an MCI diagnosis but no studies relied upon subjective memory complaints alone.

The ways in which studies identified frail and cognitively impaired groups was variable, with all except one study (Wu et al., 2020) excluding those with dementia from involvement in their study. Furthermore, four studies included those with pre-frailty in their experimental group (Kwan et al., 2019; Li et al., 2020; Rivan et al., 2020; Rivan et al., 2019).

### Psychological Factors

Data on depression or depressive symptoms were collected in all studies included in the review (see Table 3), one of these included anxiety and depression (Li et al., 2020). Three measures were used, and the Geriatric Depression Scale (GDS) (Yesavage, 1988) was collected most frequently (*n* = 7) (Aguilar-Navarro et al., 2019; Ge et al., 2020; Kwan et al., 2019; Rivan et al., 2020; Rivan et al., 2019; Shimada et al., 2016; Wu et al., 2020), the Centre for Epidemiologic Studies Depression Scale (CES-D) (Turvey et al., 1999) (*n* = 1) (Aliberti et al., 2019) and the EuroQol-5 dimension (EQ-5D) (Rabin & Charro, 2001) (*n* = 1) (Li et al., 2020) were also collected. Measures were scored with a cut-off to indicate clinical depression or not (*n* = 4) (Aliberti et al., 2019; Ge et al., 2020; Li et al., 2020; Wu et al., 2020), or summed to give a score (*n* = 5) (Aguilar-Navarro et al., 2019; Kwan et al., 2019; Rivan et al., 2020; Rivan et al., 2019; Shimada et al., 2016).

Table 4 outlines the relationships between various factors and coexistent frailty and cognitive impairment. The findings from nine studies (Aguilar-Navarro et al., 2019; Aliberti et al., 2019; Ge et al., 2020; Kwan et al., 2019; Li et al., 2020; Rivan et al., 2020; Rivan et al., 2019; Shimada et al., 2016; Wu et al., 2020) show those living with coexistent frailty and cognitive impairment have significantly higher levels of depressive symptoms than the comparison peer groups. Of particular interest is the only study which included individuals with dementia (Wu et al., 2020) with depression being far higher in those with frailty than those with pre-frailty (88% depressed with frailty vs. 56.2% depressed with pre-frailty). Additionally, further analyses uniformly reveal a significant positive association between depression and the coexistence of frailty and cognitive impairment (see Table 5).

**Table 4:** Results of Studies Examining Differences between Groups.

Factor	Citation	Statistical analysis	Finding	interpretation
Depressive Symptoms	Aguilar-Navarro, 2019^a^ Aliberti, 2018^a^ Ge, 2020^a^ Kwan, 2019^a^ Rivan, 2019^b^ Rivan, 2020^b^ Shimada, 2016^a^ Wu, 2020^c^	Chi-square Chi-square Chi-square t-test, chi-square, Mann-Whitney U t-test t-test t-test one-way ANOVA	Significant at <0.001 Significant at <0.001 Significant at <0.001 Significant at <0.001 Significant at <0.001 Significant at <0.001 Significant at <0.001 Significant at <0.001	Comorbid group greater number of symptoms than peers Comorbid group greater number of symptoms than peers Comorbid group greater number of symptoms than peers Comorbid group greater number of symptoms than peers Comorbid group greater number of symptoms than peers Comorbid group greater number of symptoms than peers Comorbid group greater number of symptoms than peers Comorbid group greater number of symptoms than peers
Depression and anxiety	Li, 2020^a^	Chi-square	Significant at <0.0001	Comorbid group report more problems than peers
Marital status	Aliberti, 2018^a^ Li, 2020^a^ Rivan, 2019^b^ Wu, 2020^c^	Chi-square Chi-square Chi-square Chi-square	Significant at <0.001 Significant at <0.0001 Not significant =0.936 Not significant +0.619	Comorbid group less likely to be married than peers Comorbid group less likely to be married than peers No difference in marital status across groups No difference in marital status across groups
Living alone status	Rivan, 2020^b^ Shimada, 2016^a^ Wu, 2020^c^	Chi-square Chi-square Chi-square	Significant at <0.05 Significant at <0.001 Not Significant =0.910	Comorbid group more likely to live alone than peers Comorbid group more likely to live alone than peers No difference in co-residence status across groups
Education level	Aliberti, 2018^a^ Kwan, 2019^a^ Li, 2020^a^ Rivan, 2019^b^ Rivan, 2020^b^ Shimada, 2016^a^ Wu, 2020^c^	Chi-square t-test, chi-square, Mann-Whitney U Chi-square Chi-square t-test t-test One-way ANOVA	Significant at <0.001 Not significant =0.053 Significant at <0.0001 Significant at <0.001 Significant at <0.001 Significant at <0.001 Not significant =0.102	Comorbid group less years in education than peers No difference in education years across groups Comorbid group less years in education than peers Comorbid group less years in education than peers Comorbid group less years in education than peers Comorbid group less years in education than peers No difference in education years across groups
Employment status	Rivan, 2019^b^	Chi-square	Not significant =0.652	No difference in employment status across groups
Net worth	Aliberti, 2018^a^	ANOVA or Kruskal-Wallis	Significant at <0.001	Comorbid group has less net worth than peers
Household income	Rivan, 2019^b^ Rivan, 2020^b^	Chi-square t-test	Significant at <0.05 Significant at =0.001	Comorbid group has less income than peers Comorbid group has less income than peers
Social Support	Rivan, 2019^b^	t-test	Significant at <0.001	Comorbid group reports less support than peers

^a^study has four groups to compare a healthy group, a frail only group, a cognitively impaired only group and a comorbid group with both frailty and cognitive impairment

^b^study has two groups to compare a healthy group and a comorbid group with both frailty and cognitive impairment

^c^study has three groups to compare one with dementia and no frailty, one with dementia and prefrailty and a comorbid group with dementia and frailty

**Table 5.** Relationships Between Various Factors and Co-Existent Frailty and Cognitive Impairment.

Factor	Citation	Statistical analysis	Finding	interpretation
Depressive Symptoms	Aguilar-Navarro, 2019 Ge, 2020 Kwan, 2019 Rivan, 2019 Rivan, 2020 Wu, 2020	Multinomial logistic regression Multinomial logistic regression Multinomial logistic regression Hierarchical binary logistic regression Stepwise binary logistic regression Multiple logistic regression	Significant association 0.007 Significant association <0.001 Adjusted model significant association = 0.002 Significant association <0.001 Significant association = 0.007 Significant association = 0.022	Greater number of depressive symptoms associated with comorbidity Presence of depression associated with comorbidity Greater number of depressive symptoms associated with comorbidity Presence of depression associated with comorbidity Greater number of depressive symptoms associated with comorbidity Greater number of depressive symptoms associated with comorbidity
Depression and anxiety	Li, 2020,	Poisson regression model	Significant association <0.001	Reporting of problems with anxiety and depression associated with comorbidity
Education level	Wu, 2020	Multiple logistic regression	Not significant association = 0.924	Years of education not associated with comorbidity
Social Support	Rivan, 2019	Hierarchical binary logistic regression	Significant association <0.001	Reported lower levels of social support associated with comorbidity

### Social Factors

A range of social variables were investigated, but with no consistency in approach and in little detail. Education was collected in seven studies, either categorizing level of education (Aliberti et al., 2019; Kwan et al., 2019; Li et al., 2020; Rivan et al., 2019) or years spent in education (Rivan et al., 2020; Shimada et al., 2016; Wu et al., 2020) (Table 3). The findings related to education were mixed (Table 4). Five studies (Aliberti et al., 2019; Li et al., 2020; Rivan et al., 2020; Rivan et al., 2019; Shimada et al., 2016) identified those living with coexistent frailty and cognitive impairment had spent less time in education than comparison groups, while two studies (Kwan et al., 2019; Wu et al., 2020) reported no difference. Only one study (Wu et al., 2020) included education in regression modelling (Table 5); this found for the population they studied, education was not associated with the coexistence of frailty and cognitive impairment.

Marital status was collected in four studies (Aliberti et al., 2019; Li et al., 2020; Rivan et al., 2019; Wu et al., 2020). Two studies (Aliberti et al., 2019; Li et al., 2020) reported higher numbers of unmarried individuals in their group of frail and cognitively impaired individuals. The remaining two (Rivan et al., 2019; Wu et al., 2020) found no significant difference. Living alone was found to be significantly more common in the comorbid group in two studies (Rivan et al., 2020; Shimada et al., 2016) while another (Wu et al., 2020) did not find this association. Lack of social support was only addressed by one study (Rivan et al., 2019), which found that lower social support was associated with the coexistence of frailty and cognitive impairment.

The impact of financial resources was investigated in three studies either by assessing net worth (Aliberti et al., 2019) or household income (Rivan et al., 2020; Rivan et al., 2019). All three studies revealed those living with coexistent frailty and cognitive impairment had consistently fewer financial resources than peers. Having a current occupation was only investigated in one study (Rivan et al., 2019); the results revealing no differences whether people were currently occupied or not between the comorbid group and the comparison group.

## Discussion

This review identified only nine studies which investigated the relationship between psychological or social factors and frailty coexistent with cognitive impairment. Relationships with depression were examined most frequently and in greatest depth. All nine studies found those with coexistent frailty and cognitive impairment were more likely to report having depressive symptoms than their peers. Social factors such as education, marital status and financial resources were rarely investigated in the same level of detail, and findings were varied on the difference between groups on these factors.

### Measures of Frailty and Cognitive Impairment

Across the included studies frailty was assessed and recorded with consistency. Despite a number of assessments available which measure frailty, only the CHS criteria (Fried et al., 2001) and the FRAIL scale (Morley et al., 2012) were used. This may be attributed to these measures being preferred forms of assessment. Those classified as pre-frail were included within the comorbid group in some studies (Kwan et al., 2019; Rivan et al., 2020; Rivan et al., 2019) but not in others (Aliberti et al., 2019; Ge et al., 2020; Li et al., 2020; Shimada et al., 2016; Wu et al., 2020). In contrast to the consistency in frailty assessment, a range of tools were used to evaluate cognitive impairment in the reviewed studies. This may be partly due to the level of inclusivity within this review, for example, Aguilar-Navarro et al. (2019) were working with a population exclusively with mild cognitive impairment and vascular burden, while (Wu et al., 2020) were investigating only those with diagnoses of dementia. The variability in selection and application of measures for cognition, and the inclusion of pre-frailty in some studies, created complexity and limitations when comparing and synthesizing findings.

### Psychological Factors

Depression was explored with the greatest consistency, frequency, and depth in the studies (Aguilar-Navarro et al., 2019; Aliberti et al., 2019; Ge et al., 2020; Kwan et al., 2019; Li et al., 2020; Rivan et al., 2020; Rivan et al., 2019; Shimada et al., 2016; Wu et al., 2020). Depressive symptom levels were variable across studies but consistently greater in the comorbid group. These findings are echoed by the findings of reviews which found depression to be associated with frailty syndrome (Soysal et al., 2017) and with cognitive impairment (da Silva et al., 2013). This suggests there may be connections between depression and later life decline, be that either cognitive or physical or both. In four of the reviewed studies (Aliberti et al., 2019; Kwan et al., 2019; Li et al., 2020; Shimada et al., 2016), there were similar levels of depressive symptoms in those solely diagnosed with frailty. However, in two others (Aguilar-Navarro et al., 2019; Ge et al., 2020) the levels of depressive symptoms were far higher in the comorbid group than any of the comparison groups. These findings may implicate increased risk in those with both physical and cognitive decline. Wu et al. (2020) reported that depression was far higher in those with frailty and dementia than those with pre-frailty and dementia. One interpretation of this could be that physical decline contributes more to low mood than cognitive impairment, although these findings are based on small numbers. Given the higher levels of depressive symptomology evidenced in the comorbid groups in the reviewed studies, this is an area which may benefit from longitudinal investigation. Additionally, all studies focussed on identifying current levels of depressive symptoms and did not consider previous bouts of depression over the life course. Moreover, some studies excluded those diagnosed with depression (Aguilar-Navarro et al., 2019; Shimada et al., 2016) or psychiatric disorders (Rivan et al., 2020; Rivan et al., 2019). It is worth noting that poor endurance or fatigue, a frailty phenotype criterion, is also a symptom of depression. There is some overlap between symptoms of both frailty and cognitive impairment, and those of depression. This may create complexity firstly in diagnosing the etiology of a symptom such as fatigue, and furthermore understanding the relationship between symptoms and different health states. A history of depression has been associated with both physical and cognitive decline in later life, suggesting a long-term relationship (Crimmins, 2020). Evidence indicates that certain individuals may be predisposed to both frailty and depression, and that there may not be a causal link between depression and frailty as formerly thought; however, this requires further investigation (Mayerl et al., 2020). As the studies included in the review primarily used cross-sectional methods, with the exception of (Rivan et al., 2020), this limits the understanding which can be gained about this complex relationship.

It has been proposed that psychological distress (predominantly depression and anxiety) may escalate the development of cognitive complaints in those with frailty (Jing et al., 2020). Therefore, psychological distress may highlight a particular risk of cognitive impairment for those with frailty. Anxiety was considered only in one study (Li et al., 2020), and then only in partnership with depression and with one single self-report question. Anxiety is worthy of greater investigation given the relationship anxiety may have with increased stress hormones such as cortisol, and their contribution to frailty syndrome (Baylis et al., 2013). There are also other psychological factors that might be pertinent to explore in people with coexistent frailty and cognitive impairment. For example, psychological resources such as self-esteem and optimism have been shown to impact positively on ageing (Dumitrache et al., 2019; Hornby-Turner et al., 2017), while positive attitudes to ageing appear to decrease the risk of frailty associated decline (Gale et al., 2018). In addition to this, affective disorders have been linked with dementia risk (da Silva et al., 2013). Further work, beyond depression, is essential in understanding the role psychological factors may play in the onset and trajectory of physical and cognitive decline.

### Social Factors

Social determinants can influence the onset and trajectory of decline in later life (Andrew et al., 2008; Crimmins, 2020). This review found that relationships between frailty coexistent with cognitive impairment and education were not consistent across studies. Other work has demonstrated that there are links between frailty development and lower education levels (Dury et al., 2017), and between impaired cognition and lower education levels (Chapko et al., 2017). Considerable variation in the educational levels of study participants was identified across the reviewed studies. However, studies which reported no differences between those with coexistent frailty and cognitive impairment and comparison groups involved participants with low levels of education or high levels of homogeneity. In one study, participants had an average of less than 5 years of schooling (Wu et al., 2020), and in another only 13% of the study population had above primary level education (Kwan et al., 2019).

Instead of purely focussing on education, greater focus should perhaps be placed on lifelong engagement with activity which involves engagement with learning across the life course. Higher childhood education level, adulthood occupation and current engagement with cognitively stimulating activity have been associated with better outcomes for older adults (Ihle et al., 2017). Implicating the benefits of continued engagement with activity that promotes thinking and learning. Such exploration would be of particular value given that current engagement with cognitively stimulating activity may prove more modifiable than educational status in childhood. While there is evidence that lower educational attainment is related to health behaviours such as smoking and alcohol consumption, there may be other ways in which education contributes to later life decline (Braveman & Gottlieb, 2014). The accumulation of wear and tear across multiple physiological systems, which is termed allostatic load, is associated with chronic exposure to stress (McEwen & Stellar, 1993). Lower educational attainment may be a source of lifelong stress, given that higher education is often associated with more lucrative and secure employment in adulthood (Braveman & Gottlieb, 2014). A longitudinal study of cohort data has identified that increased allostatic load may be a possible mechanism by which educational attainment contributes to increased frailty risk (Gale et al., 2016).

Living condition or marital status were collected in six studies (Aliberti et al., 2019; Li et al., 2020; Rivan et al., 2020; Rivan et al., 2019; Shimada et al., 2016; Wu et al., 2020). One study (Wu et al., 2020) collected both. As detailed in the results, findings were mixed across the reviewed studies, suggesting that this would benefit from more in-depth exploration. While marital status may be a proxy measure of increased practical and emotional support and financial security, this is unlikely to always be the case. In the included studies, populations were almost all married and often did not define differences in being widowed, divorced or never married. While frailty syndrome is often linked with being unmarried, potential gender differences and the possible variations between the experiences of those who have never married and those who are widowed or separated from their spouse have raised questions about why this relationship occurs (Kojima et al., 2019). Longitudinal study into the relationship between loneliness and health shows increasing loneliness with ageing and that increased loneliness is associated with poorer health. However, this may be mediated by partner status, those who experience spousal loss experience greater loneliness (Dykstra et al., 2005).

Only one study examined levels of social support, finding those living with frailty and cognitive impairment reported lower levels of support than a matched group of healthy older people, perhaps suggesting social support may offer protection against comorbid decline (Rivan et al., 2019). However, as this study employed a cross-sectional design, this may simply be support which is currently required through necessity. Social support may present as one of the few modifiable factors identified by this review. A UK-based study of cross-sectional data found that cognitive and social activity were protective of cognitive function in later life (Clare et al., 2017). Other investigation implicates the value of social ties in maintaining good health in later life, however, stipulating that the quality of such relationships may be key to this health benefit (Rook & Charles, 2017). The role, which the presence or absence, or the nature of support may play in the onset of physical and cognitive decline would benefit from longitudinal exploration.

This review found that those with both frailty and cognitive impairment had lower net worth or income. This suggests an association between affluence and poor outcomes in later life. The rate at which frailty and cognitive impairment accelerate may well be attenuated by the financial resources an individual has access to (Steptoe & Zaninotto, 2020). Financial security in later life is likely to be beneficial to health and wellbeing, given the links this has with adequate housing, use of safe outdoors environments, capacity to fund healthcare and access to leisure activities (Braveman & Gottlieb, 2014). Within this review, financial resources were shown to be impactful; however, this requires further investigation. While current wealth may be an important consideration, lifelong wealth and social standing appear to play a role in later life decline. Findings from a UK study show that paternal socioeconomic status in childhood impacts on health outcomes in later life, and this was attributed to increased levels of physiologic dysregulation in response to stressor events (Gale et al., 2016). The findings of Gale and colleagues in 2016 were consistent with work in Latin America (Alvarado et al., 2008) and Europe (Landös et al., 2019), where the consequence of childhood poverty on health was still evident in later life. While some impact may be attenuated by improving circumstances throughout adulthood understanding the long-term impact of wealth and social status requires further research.

### Strengths and Limitations

This review has many strengths, including measures of frailty and cognitive impairment enabled a wide scope of the existing literature. In addition, searching the full text of publications, rather than keyword, title or abstract search has limited the risk of overlooking a study. However, limiting searches to publications written in English will have restricted the search yield, particularly given the high number of studies identified from countries where English is not a native language. As part of the exclusion criteria for this review we excluded frailty measures that contained cognitive, psychological or social components. This was to try and ensure that there was minimal overlap between the measurement of frailty, the measurement of cognitive impairment and the outcomes of interest. Therefore, frailty indices which included outcomes of interest, such as depression, were excluded from this synthesis; symptoms of frailty and cognitive impairment are shared with conditions such as depression and may contribute to this observed close relationship. Rigour in research is a key factor in advancing knowledge, and while this review aimed to be inclusive, the moderate quality of many of the included studies limits conclusions drawn from this synthesis. However, given the emergent nature of the field, overall quality and low number of studies, inclusion was considered relevant. The current evidence base predominantly originates from Asia; research is required from other continents to ensure cross-cultural application. While the review included papers from 2001, all papers included for synthesis were published since 2016. Suggesting this topic, this is an emergent area of interest.

### Future Research and Implications for Practice

The need to investigate the characteristics of individuals living with coexistent frailty and cognitive impairment is highlighted by the cumulative effect of different deficits upon wellbeing (Avila-Funes et al., 2009). Such research is ever more pertinent in the COVID-19 era, where many older adults have been increasingly isolated, and access to healthcare and support services has been limited (Baker & Clark, 2020). While depression was investigated in the reviewed studies, there was far less consideration of the role other psychological factors may play in posing a risk or protection for decline. Turning attention towards an assets-based approach and identifying potential protective factors may lead to improved intervention development. Exploration of the lifelong psychological and social circumstances and experiences of those living with coexistent frailty and cognitive impairment may lead to a greater understanding of this comorbid decline and how it might be ameliorated.

Many of the studies included in this review were seeking to test other hypotheses, such as mortality rates. Socioeconomics and comorbid depression were often secondary analyses, examining the relationship such factors have with comorbid deterioration but with insufficient consideration of how they might influence the nature and course of decline. Furthermore, the examination of modifiable factors must be a focus of research. Physical activity levels, smoking, alcohol intake and nutrition have been reviewed extensively as modifiable risk factors in relation to frailty (Lafortune et al., 2016) and dementia (Beydoun et al., 2014). However, focussing upon behavioural factors does little to fully acknowledge the multidimensional nature of later life decline. While improving educational attainment in childhood may be a long-term goal, improving access to lifelong learning and engagement with cognitively stimulating activity may have more immediate benefits. Treatments and improved identification of depression across the life course are implicated. While other modifiable factors such as social support, access to better housing and safe outdoor spaces and timely access to health and social care require investigation with this population.

Health and social care professionals play a significant role in providing not only healthcare but also support to those who live with frailty and cognitive impairment. Many working in the field acknowledge that psychological and social factors play a role in the onset and trajectory of decline in later life (Colón-Emeric et al., 2020; Gee et al., 2019). However, there is still a heavy focus on the physiological factors which are associated with later life decline (D’Avanzo et al., 2017). Increasingly gerontologists are acknowledging the implications of life course events, social factors and psychological wellbeing (Crimmins, 2020). How far this is understood and taken forward in health and social care practice is less explored and requires investigation. A tailored approach to treating and supporting individuals, with in-depth consideration of psychological and social past and present circumstances, and multidisciplinary input, is likely to be required to enable those living with coexistent frailty and cognitive impairment to live well as they age.

## Conclusion

To conclude, this review highlights social and psychological differences between those living with coexistent frailty and cognitive impairment. The findings indicate that depression, lower education, lower material wealth and less social support are related to this comorbid state. Marital status and living alone may also play a role but this is less clear and current occupation is not shown to have an impact. The review identifies a limited number of psychological and social factors that have been explored in this area and highlights further research is required, particularly in the identification of modifiable factors. Understanding the psychological and social factors linked to coexistent frailty and cognitive impairment may aid in the identification and development of interventions to reduce the incidence of physical and cognitive decline in later life and lead to improved support for those living with coexistent frailty and cognitive decline.
